# Impaired coronary flow reserve in patients with supra-normal left ventricular ejection fraction at rest

**DOI:** 10.1007/s00259-021-05566-y

**Published:** 2022-01-06

**Authors:** Ping Wu, Xiaoli Zhang, Zhifang Wu, Huanzhen Chen, Xiaoshan Guo, Chunrong Jin, Gang Qin, Ruonan Wang, Hongliang Wang, Qiting Sun, Li Li, Rui Yan, Xiang Li, Marcus Hacker, Sijin Li

**Affiliations:** 1 Department of Nuclear Medicine, First Hospital of Shanxi Medical University, Taiyuan, Shanxi China; 2 Collaborative Innovation Center for Molecular Imaging of Precision Medicine, Taiyuan, 030001 Shanxi China; 3 Department of Nuclear Medicine, Beijing Anzhen Hospital, Capital Medical University, Beijing, China; 4 Department of Cardiology, First Hospital of Shanxi Medical University, Taiyuan, Shanxi China; 5 Key Laboratory of Cell Physiology of Ministry of Education, Shanxi Medical University, Taiyuan, Shanxi China; 6 Division of Nuclear Medicine, Department of Biomedical Imaging and Image-Guided Therapy, Medical University of Vienna, Vienna, Austria

**Keywords:** Supra-normal left ventricular ejection fraction, Coronary flow reserve, ^13^ N-Ammonia PET, Prognosis

## Abstract

**Purpose:**

Recently, a “U” hazard ratio curve between resting left ventricular ejection fraction (LVEF) and prognosis has been observed in patients referred for routine clinical echocardiograms. The present study sought to explore whether a similar “U” curve existed between resting LVEF and coronary flow reserve (CFR) in patients without severe cardiovascular disease (CVD) and whether impaired CFR played a role in the adverse outcome of patients with supra-normal LVEF (snLVEF, LVEF ≥ 65%).

**Methods:**

Two hundred ten consecutive patients (mean age 52.3 ± 9.3 years, 104 women) without severe CVD underwent clinically indicated rest/dipyridamole stress electrocardiography (ECG)-gated ^13^ N-ammonia positron emission tomography/computed tomography (PET/CT). Major adverse cardiac events (MACE) were followed up for 27.3 ± 9.5 months, including heart failure, late revascularization, re-hospitalization, and re-coronary angiography for any cardiac reason. Clinical characteristics, corrected CFR (cCFR), and MACE were compared among the three groups categorized by resting LVEF detected by PET/CT. Dose–response analyses using restricted cubic spline (RCS) functions, multivariate logistic regression, and Kaplan–Meier survival analysis were conducted to evaluate the relationship between resting LVEF and CFR/outcome.

**Results:**

An inverted “U” curve existed between resting LVEF and cCFR (*p* = 0.06). Both patients with snLVEF (*n* = 38) and with reduced LVEF (rLVEF, LVEF < 55%) (*n* = 66) displayed a higher incidence of reduced cCFR than those with normal LVEF (nLVEF, 55% ≤ LVEF < 65%) (*n* = 106) (57.9% vs 54.5% vs 34.3%, *p* < 0.01, respectively). Both snLVEF (*p* < 0.01) and rLVEF (*p* < 0.05) remained independent predictors for reduced cCFR after multivariable adjustment. Patients with snLVEF encountered more MACE than those with nLVEF (10.5% vs 0.9%, log-rank *p* = 0.01).

**Conclusions:**

Patients with snLVEF are prone to impaired cCFR, which may be related to the adverse prognosis. Further investigations are warranted to explore its underlying pathological mechanism and clinical significance.

**Supplementary Information:**

The online version contains supplementary material available at 10.1007/s00259-021-05566-y.

## Introduction

Recently, a “U” hazard ratio curve between resting left ventricular (LV) ejection fraction and prognosis has been observed in patients referred for routine clinical echocardiograms; abnormal LV ejection fraction (LVEF), not only reduced but also supra-normal LVEF (snLVEF), was correlated with adverse prognosis regardless of age, sex, or other relevant comorbidities including heart failure (HF) [1]. snLVEF is proposed as a new concept because of its newly observed long- or short-term worse outcome in patients with acute or chronic coronary syndrome with or without HF and even in those without cardiac symptoms, such as old women, hypertension, tumor, sepsis, and serious anemia [1–5]. Nevertheless, its pathological mechanism has not been clarified, which may be related to multi-factors including aortic stiffness with increasing age, cardiomyocyte hypertrophy due to enhanced afterload, increased subclinical LV mass, and the compensatory effect of the constant hyperdynamic state due to the small heart [3, 6–12].

Recently, women with snLVEF were reported a propensity towards reduced coronary flow reserve (CFR), indicating a potential mechanism of coronary microvascular dysfunction (CMD) [5]. However, that study enrolled patients with more comorbidities and unbalanced sex distribution; moreover, the utilized CFR was not corrected by the rate-pressure project (RPP) to eliminate the heterogeneity among individuals [13], all of which may limit its general applicability. Consequently, more investigations are warranted with different populations. In addition, although reduced CFR is involved in a variety of diseases [14], its overall trend along with changing LVEF has not been depicted. Obtaining such information may help to identify higher risk patients at an early stage; meanwhile, under the disappointing results of clinical trials of HF with preserved LVEF (HFpEF) to date [15], it may help to clarify the phenotype and improve the tailored management.

Accordingly, given (1) the previous preliminary results; (2) the role of CMD in the pathogenesis of HFpEF [16, 17]; and (3) the well-established predictive value of CFR for prognosis [14], we hypothesize a role of CMD in the outcome of patients with snLVEF; there may be an inverted “U” curve existing between resting LVEF and CFR. Therefore, this study aims to test the hypothesis using data of patients without known serious cardiovascular disease (CVD) who performed clinically indicated quantitative ^13^ N-ammonia positron emission tomography/computed tomography (PET/CT) scan.

## Methods

### Study population

Consecutive patients clinically indicated rest/dipyridamole-stress electrocardiography (ECG)-gated quantitative ^13^ N-ammonia PET/CT for evaluation of myocardial blood flow (MBF) and CFR between December 2015 and August 2020 at the First Hospital of Shanxi Medical University (China) were retrospectively analyzed. Inclusion criteria: (1) known or suspected CVD; (2) non-CVD with elevated cardiovascular risk referred for myocardial injury assessment. Exclusion criteria: (1) presence of acute or severe CVD including acute coronary syndrome, diagnosed HF, known coronary stenosis ≥ 50%, severe arrhythmia, diagnosed cardiomyopathy, and previous history of revascularization or cerebral stroke; (2) presence of systemic illness including known sepsis, renal disease, thyroid dysfunction, anemia, and drug addicts; (3) incomplete PET/CT data. Demographic characteristics and echocardiographic LVEF (no more than 2 months away from PET/CT) were recorded through interviews and reviews of medical records. The study conforms to the declaration of Helsinki and was approved by the hospital ethics committee. Written informed consent was provided by all patients.

### Rest/stress gated ^13^ N-ammonia PET/CT

#### Patient preparation

Patients refrained from theophylline and caffeine-containing beverages for at least 12 h and withheld medications (including beta-blockers and calcium antagonists) for at least 24 h before imaging.

#### Imaging protocol

All included subjects underwent a 1-day rest/stress protocol on a single PET/CT scanner (GE Healthcare, Discovery VCT). ^13^ N-Ammonia was injected intravenously at a dose of 700–900 MBq for standardized rest and dipyridamole stress imaging according to the American Society of Nuclear Cardiology guidelines [18]. PET images were acquired in 2D mode. The simplified retention model was used for quantification, attenuation correction by cine CT with reduced radiation dose, co-registration of PET and CT borders, and partial volume (PV) correction were performed [19]. The procedure for coronary calcification assessment was performed after PET acquisition; it would be canceled if no calcification was found in the cine CT.

#### Obtaining of quantitative parameters

Quantification of myocardial perfusion was carried out using the HeartSee software package (version 3, USA, FDA 510(k) approval K171303). Relative metrics included uptake percentage of myocardial perfusion and the total area of uptake less than 60% at rest and at stress. Absolute metrics included non-corrected and corrected resting MBF (ncrMBF and crMBF), stress MBF (sMBF), and non-corrected and corrected CFR (ncCFR and cCFR). crMBF was calculated as the ratio of ncrMBF to RPP/10,000 (RPP in units of mmHg * beats/minute) [20]. cCFR was calculated as the ratio of sMBF to crMBF. cCFR < 2.5 was defined as reduced because (1) it is the commonly used cutoff value in clinical practice [21, 22]; (2) 2.9 as the cutoff value for ^82^Rb in the extremely similar scan and processing protocol with ours [19, 23], the CFR value for ^13^ N-ammonia was reported lower than ^82^Rb [24]; and (3) it served approximately as the mean value in the current study.

LV function parameters, including end-diastolic volume (EDV), end-systolic volume (ESV), and LVEF, were analyzed using Myovation software (GE Healthcare, Xeleris) based on gated PET data. Heart rate (HR), systolic blood pressure (SBP), and diastolic blood pressure (DBP) were recorded at rest and during dipyridamole infusion (2-min intervals). Peak values were defined as the HR and BP at 7 min after the start of dipyridamole infusion. Referred to previous investigations related to snLVEF [5, 25], the small heart and heart rate reserve (HRR, a surrogate marker of an increased sympathetic outflow) were considered in our current study. The small heart was defined if ESV at rest was less than 25 mL [4]. HR response to dipyridamole was defined as HRR ([(peak HR minus rest HR)/rest HR] * 100%) to account for baseline differences [5]. The analysis of coronary calcium score was performed in the Smartscore software (version 4.0, GE Healthcare, Advanced Workstation 4.4).

### Assessment of outcomes

Follow-up was performed by review of patients’ clinical records and by phone contact with patients, their relatives, or the referring physician. All follow-ups were conducted in December 2020. Follow-up time was determined from the date of the PET/CT examination to (the first) major adverse cardiac events (MACE) or the follow-up date. In view of enrolled milder patients, MACE in this study included HF, late revascularization (over 90 days following PET/CT scan), re-hospitalization, and re-coronary angiography for any cardiac reason.

### Statistical analysis

Dose–response analyses using restricted cubic spline (RCS) functions based on a linear model were used to evaluate the association between resting LVEF and cCFR in SAS software (version 9.4, SAS Institute Inc., Cary, NC, USA). A smooth curve is plotted by using the absolute differences in cCFR for patients with different LVEF with those with reference LVEF (50th percentiles) [26].

With reference to previous thresholds [3, 5] and the quartiles of our enrolled population, patients were categorized into three groups according to resting LVEF (snLVEF (LVEF ≥ 65%); normal LVEF (nLVEF, 55% ≤ LVEF < 65%); and reduced LVEF (rLVEF, LVEF < 55%)). SPSS (version 24.0. IBM Corp. Armonk, NY) was used for the following statistical analyses. For continuous variables, data were presented as mean ± standard deviation (SD) or median (interquartile range), and for categorical variables as frequency and percentage. Demographic characteristics, parameters of quantitative perfusion, LV function, and hemodynamic changes were compared among the three groups using Student’s *t*-test, Mann–Whitney test, analysis of variance (ANOVA), Kruskal–Wallis test, or chi-square tests. Prior to analyses, basic assumptions were checked, and multicollinearity testing was performed for potentially interrelated variables. Multivariable logistic regression analyses using the LR forward method were performed to identify predictors of reduced cCFR, adjusting for variables showing a *p-*value ≤ 0.11 in univariate analysis; variables were transformed into dichotomous or trichotomous variables if needed. Event-free survival curves for MACE were generated by the Kaplan–Meier method and compared by log-rank test. Patients with both PET/CT and echocardiographic LVEF detection were included in a sub-cohort analysis. Pearson correlation analysis and comparison among groups were conducted. A two-tailed *p-*value of less than 0.05 was considered statistically significant; *p* values were corrected by the Bonferroni method for pairwise comparisons among the three groups.

## Results

### Patient characteristics

Finally, 23 patients were excluded, including 4 with previous revascularization history, 1 with hypertrophic cardiomyopathy, 1 with dilated cardiomyopathy, 5 with arrhythmia, 4 with thyroid dysfunction, 1 with drug addiction, and 7 with incomplete data. As shown in Table 1, a total of 210 patients (mean age 52.3 ± 9.3 year) were finally enrolled, including 49.5% of women (*n* = 104), 52.4% of known or suspected CVD (*n* = 110), and 49.5% of abnormal LVEF (*n* = 104). Patients with CVD encountered a higher proportion of snLVEF than those without CVD (24.5% (*n* = 27) vs 11% (*n* = 11), *χ*^2^ = 6.49, *p* < 0.05); male and female had similar proportions (19.8% vs 16.3%, *χ*^2^ = 0.43, *p* > 0.05). Patients with snLVEF underwent more coronary morphological evaluation within 90 days than the other two groups (both *p* < 0.05). No differences were observed in body mass index, dyslipidemia, cardiovascular family history, coronary calcium score ≥ 100, symptoms, and medications among groups (*p* > 0.05). Ten patients were found with regionally mild thickened walls by echocardiography, mainly in the septal wall. Among whom, 2/10 were visible on PET images but with normal LVEF and cCFR, 6/10 were with snLVEF, and 2/6 were detected with reduced cCFR.

**Table 1:** Demographic characteristics of study cohort among the three LVEF groups

	Total *n* = 210	LVEF			
		< 55% (*n* = 66)	55 ~ 65% (*n* = 106)	≥ 65% (*n* = 38)	*p* value
Male, *n* (%)	106 (50.5)	37 (56.1)	48 (45.3)	21 (55.3)	0.314
Age (years)	52.3 ± 9.3	50.2 ± 9.3^a*^	52.8 ± 9.0^a,b^	54.8 ± 9.1^b^	0.038
Body mass index (kg/m^2^)	25.5 ± 3.6	25.4 ± 3.5	25.0 ± 3.8	26.7 ± 3.7	0.115
Hypertension, *n* (%)	86 (42.4)	19 (29.2)^a^	43 (42.6)^a,b^	24 (64.9)^b^	0.002
Diabetes, *n* (%)	49 (24.4)	15 (23.4)	23 (22.8)	11 (30.6)	0.632
Dyslipidemia, *n* (%)	92 (45.5)	32 (49.2)	41 (41.0)	19 (51.4)	0.429
Smoking, *n* (%)	72 (34.4)	20 (30.3)	35 (33.3)	17 (44.7)	0.310
Cardiovascular family history, *n* (%)	40 (18.8)	15 (21.7)	19 (17.6)	6 (16.7)	0.939
Coronary calcium score ≥ 100, *n* (%)	27 (12.9)	6 (9.1)	13 (12.3)	8 (21.1)	0.207
Coronary morphological examination within 90 days, *n* (%)	103 (49.0)	28 (42.4)^a^	47 (44.3)^a^	28 (73.7)^b^	0.003
Angina pectoris and dyspnea, *n* (%)	21 (10.0)	7 (10.6)	8 (7.5)	6 (15.8)	0.154
Cardiovascular risk number^†^	4 (2.6)	4 (2.5)^a^	4 (2.5)^a^	6 (4.7)^b^	< 0.001
Patients with thickened wall	10 (4.8%)	1 (1.5%)	3 (2.8%)	6(15.8%)	
Medication, *n* (%)	104 (49.6)	29 (43.9)	54 (50.9)	21 (55.3)	0.494
Calcium channel blocker	56 (26.7)	17 (25.8)	26 (24.5)	13 (34.2)	
Statin	45 (21.4)	20 (30.3)	18 (17.0)	7 (18.4)	
Hypoglycemic	28 (13.3)	6 (9.1)	17 (16.0)	5 (13.2)	
Platelet inhibitor	27 (12.9)	11 (16.7)	11 (10.4)	5 (13.2)	
β blocker	18 (8.6)	5 (7.6)	10 (9.4)	3 (7.9)	
ACEI or ARB	17 (8.1)	7 (10.6)	9 (8.5)	1 (2.6)	
Nitrate	9 (4.3)	0	6 (5.7)	3 (7.9)	
Anti-ischemic metabolism^‡^	6 (2.9)	3 (4.5)	2 (1.9)	1 (2.6)	
MACE, *n* (%)	8 (4.2)	3 (4.5)^a,b^	1 (0.9)^a^	4 (10.5)^b^	0.036

^*^Statistically significant difference between letters

^†^The sum of risk factor scores including age > 55 years, female, body mass index > 25 kg/m^2^, hypertension, hyperlipidemia, diabetes, smoke, cardiovascular family history, coronary calcium score ≥ 100, coronary morphological examination within 90 days and symptoms, one point for each risk factor

^‡^Include trimetazidine, renolazine, and vansolil

*ACEI*, angiotensin-converting enzyme inhibitor; *ARB*, angiotensin receptor blocker; *LVEF*, left ventricular ejection fraction; *MACE*, major adverse cardiac events

### The association between resting LVEF and cCFR

**Fig. 1:**
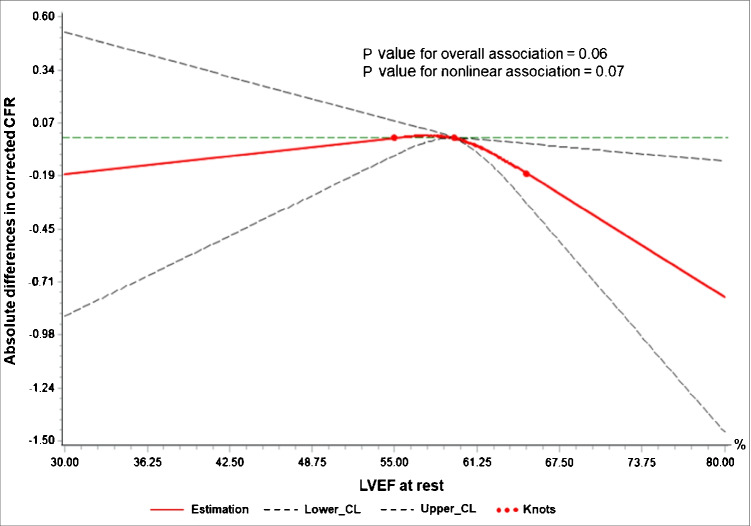
The dose–response association between resting LVEF and cCFR. The LVEF in X-axis is coded with three knots (red dots) located at 55%, 60% (50th percentiles, as the reference), and 65%. The *Y*-axis represents the differences in cCFR between patients with any value of LVEF with those with 60%. The red solid line depicts the smooth curve of the differences. The horizontal green dashed reference line is displayed to materialize the null hypothesis H0. The black dashed lines located on the same side of the reference line in snLVEF segment stand for statistically significant 95% confidence intervals (CI). CL, confidence limits; cCFR, corrected coronary flow reserve; LVEF, left ventricular ejection fraction

As illustrated in Fig. 1, taking 60% of LVEF as the reference, a roughly inverted “U” smooth curve (red solid line) is plotted to visualize the association between resting LVEF and cCFR. Compared to patients with nLVEF, patients with snLVEF and rLVEF displayed bigger negative differences (indicating decreased cCFR), especially the former. The slope in the snLVEF segment goes steeply downwards with statistically significant 95% confidence intervals (CI) (black dashed lines located on the same side of the green reference line), and the slope in the rLVEF segment goes gently with non-significant 95% CI, finally leading to a *p*-value higher than 0.05 in the overall curve (overall association: *χ*^2^ = 5.55, *p* = 0.06; nonlinear association: *χ*^2^ = 3.29, *p* = 0.07).

### Comparison findings of ^13^ N-ammonia PET/CT among the three groups

**Fig. 2:**
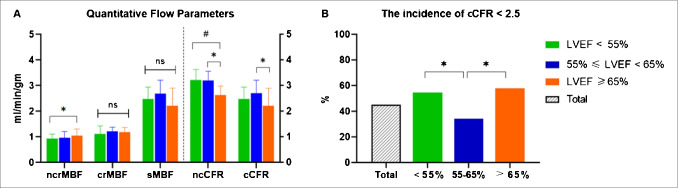
^13^ N-Ammonia quantitative findings among the three LVEF groups. **A** Quantitative flow parameters. **B** The incidence of reduced cCFR. *p < 0.05, #*p* < 0.001, *p-*value between different LVEF groups is corrected by the Bonferroni method, ns represents not significant. CFR, coronary flow reserve; LVEF, left ventricular ejection fraction; MBF, myocardial blood flow; lowercase letter before uppercase parameters: c, corrected; nc, non-corrected; r, rest; s, stress

As demonstrated in Table 2, the snLVEF group had significantly lower ncCFR and cCFR than the nLVEF group (*p* < 0.001 and *p* < 0.05, respectively, Fig. 2A). ncrMBF and sMBF in the snLVEF group did not differ significantly from those in the nLVEF group (both *p* > 0.05, Fig. 2A), while the medians were 8.2% higher and 7.9% lower, respectively. crMBF did not differ among the three groups (*p* > 0.05, Fig. 2A). Both the snLVEF and the rLVEF group displayed a higher incidence of reduced cCFR than the nLVEF group (both *p* < 0.05, Fig. 2B). The snLVEF group also displayed more small hearts (*p* < 0.001). The rLVEF group displayed significantly enlarged ESV (both *p* < 0.001 at rest and stress) and EDV (*p* < 0.05 at rest, and *p* ≤ 0.001 at stress, respectively). HR, SBP, and DBP at rest and stress as well as HRR did not differ among the three groups (*p* > 0.05); the mean value of HRR in the snLVEF group was 15.4% lower than that in the nLVEF group.

**Table 2:** Comparison of findings in ^13^ N-ammonia PET/CT among the three LVEF groups

	Total *n* = 210	LVEF			*p* value
		< 55% (*n* = 66)	55–65% (*n* = 106)	≥ 65% (*n* = 38)	
*Absolute perfusion*					
ncrMBF (mL/min/g)	0.97 (0.82, 1.18)	0.93 (0.76, 1.11)^a^	0.97 (0.85,1.21)^a,b^	1.05 (0.91, 1.31)^b^	0.018
crMBF (mL/min/g)	1.17 (1.01, 1.41)	1.12 (0.97, 1.43)	1.22 (1.04, 1.38)	1.18 (1.04, 1.37)	0.564
sMBF (mL/min/g)	2.97 (2.35, 3.71)	2.84 (2.32, 3.39)	3.16 (2.65, 3.93)	2.91 (2.14, 3.61)	0.073
ncCFR	3.06 (2.56, 3.52)	3.21 (2.56, 3.63)^a^	3.20 (2.65, 3.56)^a^	2.63 (2.23, 2.98)^b^	< 0.001
cCFR	2.63 ± 0.74	2.56 ± 0.72^a,b*^	2.76 ± 0.72^a^	2.41 ± 0.75^b^	0.027
cCFR < 2.5, *n* (%)	94 (45.0)	36 (54.5)^a^	36 (34.3)^b^	22 (57.9)^a^	0.007
*Relative perfusion*					
rUPTAKE (%)	80 ± 3	80 ± 4	81 ± 2	78 ± 3	0.189
sUPTAKE (%)	80 ± 3	81 ± 4	79 ± 3	79 ± 3	0.742
Abnormal area at rest (%^†^)	6 ± 5	7 ± 5	6 ± 5	5 ± 4	0.327
Abnormal area at stress (%^†^)	7 ± 5	8 ± 6	7 ± 5	7 ± 5	0.247
*Hemodynamic changes during dipyridamole stress*					
rHR (bpm)	68 ± 11	66 ± 12	69 ± 10	69 ± 12	0.101
sHR (bpm)	92 ± 14	92 ± 13	94 ± 15	91 ± 12	0.296
HRR (%)	38 ± 20	41 ± 21	39 ± 20	33 ± 15	0.100
rSBP (mmHg)	128 ± 18	125 ± 19	127 ± 18	133 ± 16	0.100
sSBP (mmHg)	122 (111, 130)	116 (106, 128)	121 (111, 131)	126 (114, 134)	0.100
rDBP (mmHg)	72 (64,81)	70 (61,80)	70 (62, 80)	75 (68, 86)	0.070
sDBP (mmHg)	67 ± 11	67 ± 12	66 ± 10	68 ± 10	0.689
rRPP (mmHg * bpm)	8639 ± 1905	8181 ± 1804^a^	8706 ± 1810^a,b^	9243 ± 2168^b^	0.021
sRPP (mmHg * bpm)	11,316 ± 3179	11,155 ± 4402	11,369 ± 2427	11,449 ± 2486	0.878
*Left ventricular function*					
rEDV (mL)	84 (73, 102)	97 (83, 111)^a^	78 (71, 95)^b^	83 (70, 96)^b^	< 0.001
rESV (mL)	34 (29, 44)	46 (39, 53)^a^	32 (28, 37)^b^	26 (22, 32)^c^	< 0.001
Small heart	30 (14.3)	0^a^	13 (12.3%)^b^	17 (44.7)^c^	< 0.001
Rest LVEF (%)	60 (54,63)	53 (50, 54)^a^	60 (58, 62)^b^	67 (66, 70)^c^	< 0.001
sEDV (mL)	94 (82, 113)	108 (90, 124)^a^	89 (79, 106)^b^	89 (75, 105)^b^	< 0.001
sESV (mL)	32 (26, 40)	40 (33, 46)^a^	30 (26, 35)^b^	29 ± 8^b^	< 0.001
sLVEF (%)	66 (62, 69)	63 (57, 65)^a^	67 (64, 70)^b^	70 (67, 72)^b^	< 0.001

^*^Statistically significant difference between letters

^†^Area of uptake% less than 60%

*CFR*, coronary flow reserve; *DBP*, diastolic blood pressure; *EDV*, end-diastolic volume; *ESV*, end-systolic volume; *HR*, heart rate; *HRR*, heart rate reserve; *LVEF*, left ventricular ejection fraction; *MBF*, myocardial blood flow; *PET/CT*, positron emission tomography/computed tomography; *RPP*, rate-pressure product; *SBP*, systolic blood pressure; lowercase letter before uppercase parameters: *c*, corrected; *nc*, non-corrected; *r*, rest; *s*, stress

### Risk predictors of reduced cCFR

After adjusting by age, sex, hypertension, sMBF, rSBP, rDBP, sSBP, rRPP, rESV (trichotomous variable), rEDV (dichotomous variable), sESV (dichotomous variable), sEDV (dichotomous variable), and HRR (dichotomous variable), regression analysis results revealed that both snLVEF and rLVEF remained independent predictors for reduced cCFR (*p* < 0.01 and *p* < 0.05, respectively, Table 3). Area under curve of the receiver operating characteristic curve (ROC) of the output model was 0.85 (95% CI: 0.80–0.90), *p* < 0.001; the sensitivity was 90.1%; and the specificity was 66.1%.

**Table 3:** Multivariate regression analysis for the impact of LVEF on reduced cCFR

	OR	95% CI	*p* value
Supra-normal LVEF	4.20	1.53–11.54	0.005
Reduced LVEF	2.46	1.12–5.41	0.025
Female * blunted HRR	3.57	1.25–10.15	0.017
rEDV > 83 mL	3.31	1.69–6.46	< 0.001
Age	1.09	1.05–1.13	< 0.001
sMBF	0.41	0.28–0.59	< 0.001
rRPP	0.999	0.999–1.000	< 0.001

*cCFR*, corrected coronary flow reserve; *CI*, confidence interval; *rEDV*, end-diastolic volume at rest; *HRR*, heart rate reserve; *LVEF*, left ventricular ejection fraction; *sMBF*, myocardial blood flow at stress; *OR*, odds ratio; *rRPP*, rate-pressure product at rest

### Follow-up results and preliminary survival analysis

**Fig. 3:**
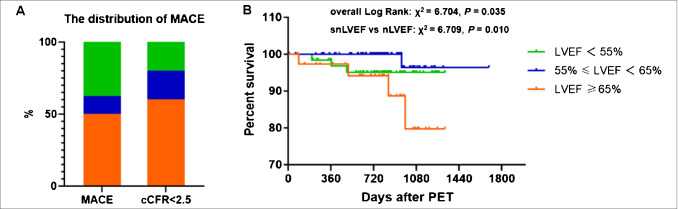
The distribution of patients incurred MACE and MACE with reduced cCFR (**A**) and the Kaplan–Meier curves (**B**) according to the three LVEF groups. cCFR, corrected coronary flow reserve; LVEF, left ventricular ejection fraction; MACE, major adverse cardiac events; PET, positron emission tomography

Patients were followed for 27.3 ± 9.5 months (range 3.4–56.4 months). Eight patients experienced MACE, including 3 percutaneous coronary interventions, 1 HF, 3 re-hospitalizations, and 1 re-coronary angiography due to chest pain. Of those, 5 (62.5%) had reduced cCFR, 4 (50%) had snLVEF or were female, and 3 (37.5%) had rLVEF or small hearts. MACE differed significantly only between snLVEF and nLVEF groups (*p* < 0.01, Table 1). Patients with both normal LVEF and normal cCFR did not incur any MACE. Figure 3 depicts the distribution of MACE and MACE with reduced cCFR in the three LVEF groups (Fig. 3A), and the Kaplan–Meier curves (Fig. 3B). MACE-event-free survival in patients with snLVEF was significantly lower than that in patients with nLVEF (*χ*^2^ = 6.71, log-rank *p* = 0.01).

### Sub-cohort analysis according to echocardiographic LVEF

There was a good correlation between PET/CT and echocardiographic LVEF values (*n* = 151, *r* = 0.627, *p* < 0.001). There was no significant difference among the three groups for demographic characteristics (Supplementary Table 1). The snLVEF group had 8% lower the value of cCFR, 62.5% higher the incidence of cCFR reduction, and 1.2 times higher the risk of MACE than the nLVEF group (Supplementary Tables 1 and 2). After adjustment for the same variables in the regression analysis, snLVEF was still an independent variable for cCFR reduction (Supplementary Table 3).

## Discussion

Patients with resting snLVEF have recently stood out because of their worse prognosis in contrast to patients with nLVEF, even if patients without HF [1, 4, 5]. However, the pathogenesis has not yet been well-elucidated. Building on previous investigations, we explored the relationship between resting LVEF detected by PET/CT and CFR as well as the role of CMD in the adverse outcome of patients with snLVEF; LVEF at stress was not considered due to its limited accessibility in clinical practice. In our milder cohort without severe CVD, CFR in the snLVEF group displayed a stable difference when compared to the nLVEF group, regardless of continuous/binary or corrected/non-corrected. Patients with snLVEF had over 4 times and almost 2 times the risk of reduced cCFR than those with nLVEF and rLVEF, respectively. To our best knowledge, this is the first study to depict the trend of cCFR with changing LVEF at rest, an inverted “U” curve was found between the two, with a peak at 55–65%, and the slope of our plotted curve coincided with the aforementioned LVEF-prognosis curve reported recently in a large-sample clinical study including 203,135 patients [1]. Finally, our preliminary small-sample follow-up results also observed a higher risk of MACE in patients with snLVEF. These interesting findings suggest to some extent the potential prognostic value of CMD in patients with snLVEF. Our study provided further evidence to support the potential clinical and scientific value of snLVEF.

Of note, the odds ratio value of snLVEF predicting reduced CFR in the current study was lower than that in the previous study [5] (4.2 vs 6.9), which might relate to the milder conditions of enrolled patients; meanwhile, the milder conditions might also diminish the recognized severity of patients with rLVEF in our study [27]. As for the worse of patients with snLVEF than those with rLVEF, the accompanying more risk factors might principally contributed, additionally, higher attention and thereby appropriate management of patients with rLVEF in practice might provide a further interpretation [27].

Limited literature reported the pathomechanism of reduced CFR in patients with snLVEF. Neurovascular hyperactivity at rest and inadequate reserve at stress were speculated preliminarily as one mechanism, reflecting with decreased HRR, higher ncrMBF, and thus reduced ncCFR [5, 25, 28]. However, ncrMBF without correction by RPP would be influenced by the physiological response and disturb the interpretation of the results [13]. Currently, no literature reports the change of crMBF in patients with snLVEF. In our study, patients with snLVEF displayed higher ncrMBF and blunted HRR (albeit with no statistical difference); nevertheless, the crMBF turned to be closer. Therefore, the observed CMD (reduced CFR) probably represented a true pathological change associated with the adverse outcome of patients with snLVEF. Secondly, the hyperdynamic workload in patients with snLVEF upregulates cardiomyocyte oxygen demand, which may result in ensuing microvascular ischemia, myocardial injury, interstitial fibrosis, and impaired cardiac mechanics, and thus finally representing by the detected reduction of CFR [17]. Lastly, consistent with previous studies [3, 11, 12], patients with snLVEF in the present study incurred more comorbidities, which may lead to chronic systemic low-grade inflammation, resulting in myocardial remodeling and dysfunction via the endothelium-cardiomyocyte signaling [29]. Furthermore, a higher comorbidity burden may induce emotional stress, contributing to CMD via the neuro-inflammatory-vascular circuit [30, 31].

The incidence of snLVEF was reported to be 22 ~ 33% in previous similar studies [4, 5] and 11.3% in patients in intensive care units [3]. It reached 18.1% in our total cohort and accounted for a notable proportion of 11.0% in non-CVD risk populations. Given the high risk and incidence of snLVEF, further prospective studies designed with a long-term follow-up, multi-functional molecular imaging with neurobiological evaluation may provide an interrelating insight in the future.

It is worth noticing that the consistency of functional parameters in this study was not verified by magnetic resonance imaging or echocardiography except for a good correlation of LVEF between PET and echocardiography in the sub-cohort analysis. Despite patients with echocardiographic snLVEF also displayed a higher incidence of CFR reduction, and snLVEF remained the independent predictor for reduced CFR after adjustment for multivariable factors, some deficiencies such as the limited sample size, the time interval between echocardiography and PET/CT, and their technological difference in LVEF detection would cause statistical bias; therefore, further prospective investigations are warranted. On the other hand, the quantification of cardiac PET suffers from position- and time-dependent PV effect in addition to the tracer, the vasodilator, the protocol, and the tissue-compartment model [14, 23, 32–34]. A marked thickened wall or small LV cavity may overestimate LVEF due to the inaccurate delineation of the endocardium in systole [33]. However, literature proved good correlations among the three imaging modalities [34], and ^13^ N-ammonia utilized in the current study has a higher image quality and a less PV effect due to its shorter positron range than ^82^Rb [21, 23]; additionally, considerable efforts have been expended to increase the reliability of our results, such as correct co-registration, PV correction by established factors, exclusion of diseases that might interfere with the results, and consideration of LV volume into the regression model. The 10 cases of mildly thickened walls in the current study would merely yield negligible influence. Nonetheless, the conclusions in the present study are currently mainly applied to PET.

There were some other limitations to this study. First, it was a retrospective study with a potential selection bias, which may lead to the type I error, and, meanwhile, limit causal inference. Second, the limited sample size restrains further analysis of subgroup comparisons (such as normal vs abnormal CFR, male vs female) in patients with snLVEF. Third, although patients with known obstructive stenosis were excluded, approximately half of patients did not undergo evaluation of coronary stenosis; their coronary conditions were unknown; however, the calcification evaluation was done in all patients, which can partly serve as an inspector in the current low-risk cohort [35].

## Conclusions

An inverted “U” curve between resting LVEF and cCFR was found in patients without severe CVD who underwent clinically indicated ^13^ N-ammonia PET/CT, and patients with snLVEF are prone to impaired cCFR, which may be related to the adverse prognosis. Further prospective investigations are warranted to explore its underlying pathological mechanism and clinical significance.

## Supplementary Information

Below is the link to the electronic supplementary material.

Supplementary file1 (DOCX 30 KB)

## Data Availability

The dataset generated and analyzed during this study is available from the corresponding author on reasonable request.
