# Supplementary material for: Impaired coronary flow reserve in patients with supra-normal left ventricular ejection fraction at rest
Source: Eur J Nucl Med Mol Imaging. 2022 Jan 6;49(7):2189–98. doi: 10.1007/s00259-021-05566-y (PMC9165269; doi:10.1007/s00259-021-05566-y)
Supplement: Supplementary file 1 — Supplementary file1 (DOCX 30 KB) [file 259_2021_5566_MOESM1_ESM.docx]

**Supplementary materials for sub-cohort analysis categorized by echocardiographic LVEF**

**TABLE** **1** Demographic characteristics categorized by echocardiographic LVEF

|  | Total  n = 151 | LVEF | | | |
| --- | --- | --- | --- | --- | --- |
|  |  | < 55%  (n = 11) | 55%-65%  (n = 65) | ≥65%  (n = 75) | *p* value |
| Male, n (%) | 71(47.0) | 7(63.6) | 30(46.2) | 34(45.3) | 0.516 |
| Age (years) | 52.5±9.4 | 49.6±9.4 | 51.5±9.5 | 53.8±9.2 | 0.211 |
| Body mass index (kg/m^2^) | 25.2±3.8 | 25.0±4.6 | 25.8±3.8 | 24.8±3.6 | 0.272 |
| Hypertension, n (%) | 71(47.0) | 5(45.5) | 31(48.4) | 35(48.6) | 0.981 |
| Diabetes, n (%) | 38(25.2) | 4(36.4) | 18(29.0) | 16(22.5) | 0.514 |
| Dyslipidemia, n (%) | 77(51.0) | 2(18.2) ^a*^ | 40(63.5) ^b^ | 35(48.6) ^a,b^ | 0.013 |
| Smoking, n (%) | 48(31.8) | 5(45.5) | 17(26.2) | 26(34.7) | 0.335 |
| Cardiovascular family history, n (%) | 25(16.6) | 2(18.2) | 8(12.9) | 15(21.1) | 0.457 |
| Coronary calcium score ≥ 100, n (%) | 18(11.9) | 0(0) | 7(10.8) | 11(14.7) | 0.348 |
| Coronary morphological examination within 90 days, n (%) | 89(58.9) | 6(54.5) | 44(67.7) | 39(52.0) | 0.162 |
| Angina pectoris and dyspnea, n (%) | 19(12.6) | 2(18.2) | 9(13.8) | 8(10.7) | 0.720 |
| Cardiovascular risk number ^†^ | 5(3,6) | 3(2,7) | 5(3,7) | 5(3,6) | 0.383 |
| Medication‡, n (%) | 80(53.0) | 3(27.3) | 34(52.3) | 43(57.3) | 0.494 |
| MACE, n (%) | 7(4.6) | 0(0) | 2(3.1) | 5(6.7) | 0.356 |

* Statistically significant difference between letters. † represents the sum of risk factor scores including age > 55y, female, body mass index > 25kg/m^2^, hypertension, hyperlipidemia, diabetes, smoke, cardiovascular family history, coronary calcium score ≥ 100, coronary morphological examination within 90 days and symptoms, one point for each risk factor. ‡ Medication represents total utilization of calcium channel blocker, statin, hypoglycemic, platelet inhibitor, βblocker, ACEI (angiotensin-converting enzyme inhibitor), ARB (angiotensin receptor blocker), nitrate, and anti-ischemic metabolism (including trimetazidine, renolazine and vansolil). LVEF, left ventricular ejection fraction; MACE, major adverse cardiac events.

**TABLE 2** Comparison of ^13^N-ammonia PET/CT findings categorized by echocardiographic LVEF

|  | Total  n = 151 | LVEF | | | *p* value |
| --- | --- | --- | --- | --- | --- |
|  |  | < 55%  (n = 11) | 55%-65%  (n = 65) | ≥65%  (n = 75) |  |
| *Absolute perfusion* |  |  |  |  |  |
| ncrMBF(mL/min/g) | 0.97(0.82,1.18) | 0.93(0.76,1.11) | 0.97(0.85,1.21) | 1.05(0.91,1.31) | 0.527 |
| crMBF (mL/min/g) | 1.16(1.00,1.34) | 1.12(0.97,1.43) | 1.22(1.04,1.38) | 1.18(1.04,1.37) | 0.482 |
| sMBF (mL/min/g) | 3.06(2.34,3.72) | 2.84(2.32,3.39) | 3.16(2.65,3.93) | 2.91(2.14,3.61) | 0.478 |
| ncCFR | 3.06(2.59,3.52) | 3.21(2.56,3.63) | 3.20(2.65,3.56) | 2.63(2.23,2.98) | 0.325 |
| cCFR | 2.67±0.75 | 2.47±0.86 | 2.80±0.71 | 2.58±0.76 | 0.169 |
| cCFR<2.5, n (%) | 62(41.3) | 7(63.6) ^a*^ | 19(29.7) ^b^ | 36(48.0) ^a^ | 0.026 |
| *Relative perfusion* |  |  |  |  |  |
| rUPTAKE, % | 80±3 | 80±4 | 81±3 | 78±3 | 0.183 |
| sUPTAKE, % | 80±4 | 81±4 | 80±4 | 79±3 | 0.361 |
| abnormal area at rest, %^†^ | 6±5 | 8±6 | 6±5 | 6±4 | 0.361 |
| abnormal area at stress, %^†^ | 7±5 | 9±6 | 8±6 | 7±5 | 0.490 |
| *Hemodynamic changes during dipyridamole stress* | | | | | |
| rHR (bpm) | 68±11 | 69±10 | 68±10 | 68±11 | 0.931 |
| sHR(bpm) | 93±13 | 99±16 | 93±11 | 91±13 | 0.142 |
| HRR (%) | 38±17 | 44±16 | 39±16 | 36±18 | 0.315 |
| rSBP(mmHg) | 130±18 | 120±18 | 131±18 | 129±18 | 0.149 |
| sSBP (mmHg) | 123(111,132) | 127(96,136) | 123(113,134) | 122(108,130) | 0.308 |
| rDBP (mmHg) | 73(66,81) | 81(64,86) | 72(66,81) | 73(64,81) | 0.391 |
| sDBP (mmHg) | 67±10 | 65±14 | 68±10 | 66±11 | 0.433 |
| rRPP(mmHg*bpm) | 8775±1959 | 8282±1937 | 8878±1915 | 8760±2014 | 0.648 |
| sRPP(mmHg*bpm) | 11416±3427 | 14466±9418 | 11591±1882 | 10812±2608 | 0.051 |

*Statistically significant difference between letters. † Area of uptake% less than 60%.

CFR, coronary flow reserve; DBP, diastolic blood pressure; EDV, end-diastolic volume; ESV, end-systolic volume; HR, heart rate; HRR, heart rate reserve; LVEF, left ventricular ejection fraction; MBF, myocardial blood flow; PET/CT, positron emission tomography/computed tomography; RPP, rate-pressure product; SBP, systolic blood pressure; lowercase letter before uppercase parameters c, corrected; nc, non-corrected; r, rest; s, stress.

**TABLE 3** Multivariate regression analysis for predictors of reduced cCFR

|  | OR | 95% CI | *p* value |
| --- | --- | --- | --- |
| Supra-normal LVEF | 2.69 | 1.10-6.60 | 0.031 |
| Reduced LVEF | 5.76 | 1.19-27.95 | 0.030 |
| Female* Blunted HRR | 4.67 | 1.28-17.07 | 0.020 |
| sEDV > 94ml | 3.22 | 1.42-7.33 | 0.005 |
| Age | 1.09 | 1.05-1.14 | <0.001 |
| sMBF | 0.318 | 0.192-0.526 | <0.001 |
| rRPP | 0.999 | 0.999-1.000 | <0.001 |

cCFR, corrected coronary flow reserve; CI, confidence interval; sEDV, end-diastolic volume at stress; HRR, heart rate reserve; LVEF, left ventricular ejection fraction; sMBF, myocardial blood flow at stress; OR, odds ratio; rRPP, rate-pressure product at rest.
